# Advanced Respiratory Monitoring during Extracorporeal Membrane Oxygenation

**DOI:** 10.3390/jcm13092541

**Published:** 2024-04-26

**Authors:** Rachele Simonte, Gianmaria Cammarota, Luigi Vetrugno, Edoardo De Robertis, Federico Longhini, Savino Spadaro

**Affiliations:** 1 Department of Medicine and Surgery, Università degli Studi di Perugia, 06100 Perugia, Italy; rachele.simonte@gmail.com (R.S.); edoardo.derobertis@unipg.it (E.D.R.); 2 Department of Translational Medicine, Università del Piemonte Orientale, 28100 Novara, Italy; gmcamma@gmail.com; 3 Department of Medical, Oral and Biotechnological Sciences, University of Chieti-Pescara, 66100 Chieti, Italy; luigi.vetrugno@unich.it; 4 Department of Medical and Surgical Sciences, Università della Magna Graecia, 88100 Catanzaro, Italy; 5 Anesthesia and Intensive Care Unit, “R. Dulbecco” University Hospital, 88100 Catanzaro, Italy; 6 Department of Morphology, Surgery and Experimental Medicine, University of Ferrara, 44100 Ferrara, Italy; savinospadaro@gmail.com

**Keywords:** respiratory monitoring, ECMO, acute respiratory failure, electrical impedance tomography, esophageal pressure, transpulmonary pressure, lung ultrasound, diaphragm ultrasound, electrical activity of the diaphragm

## Abstract

Advanced respiratory monitoring encompasses a diverse range of mini- or noninvasive tools used to evaluate various aspects of respiratory function in patients experiencing acute respiratory failure, including those requiring extracorporeal membrane oxygenation (ECMO) support. Among these techniques, key modalities include esophageal pressure measurement (including derived pressures), lung and respiratory muscle ultrasounds, electrical impedance tomography, the monitoring of diaphragm electrical activity, and assessment of flow index. These tools play a critical role in assessing essential parameters such as lung recruitment and overdistention, lung aeration and morphology, ventilation/perfusion distribution, inspiratory effort, respiratory drive, respiratory muscle contraction, and patient–ventilator synchrony. In contrast to conventional methods, advanced respiratory monitoring offers a deeper understanding of pathological changes in lung aeration caused by underlying diseases. Moreover, it allows for meticulous tracking of responses to therapeutic interventions, aiding in the development of personalized respiratory support strategies aimed at preserving lung function and respiratory muscle integrity. The integration of advanced respiratory monitoring represents a significant advancement in the clinical management of acute respiratory failure. It serves as a cornerstone in scenarios where treatment strategies rely on tailored approaches, empowering clinicians to make informed decisions about intervention selection and adjustment. By enabling real-time assessment and modification of respiratory support, advanced monitoring not only optimizes care for patients with acute respiratory distress syndrome but also contributes to improved outcomes and enhanced patient safety.

## 1. Introduction

Hypoxemic acute respiratory failure (ARF) and acute respiratory distress syndrome (ARDS) stand out as well-recognized causes of intensive care unit (ICU) admissions [1]. In patients with severe ARDS and refractory respiratory failure, veno-venous extracorporeal membrane oxygenation (vv-ECMO) assumes a critical role in their care [2]. During vv-ECMO, blood is drained through a venous cannula commonly positioned in the femoral vein. Blood is oxygenated by an artificial membrane lung and subsequently reinfused through another (reinfusion) cannula, positioned in the femoral or jugular vein. Several trials have demonstrated encouraging outcomes and benefits in favor of vv-ECMO in the most severe ARDS cases of varying etiology [3,4,5,6,7].

When devising a respiratory support strategy, it is imperative to consider both the severity and history of the underlying lung condition. Furthermore, it is essential to be mindful of potential harm that could be inflicted on the lungs and respiratory muscles due to improper management of mechanical ventilation. It should be emphasized that injuries to these components may arise either during spontaneous breathing or be provoked by inadequately configured ventilator parameters [8,9]. Currently, clinicians can utilize ventilator-assessed pressures to ensure protective mechanical ventilation, with the aim of mitigating ventilator-induced lung injury [10]. Strategies targeting the reduction of these adverse effects focus on assessing and decreasing total stress (transpulmonary pressure) and strain (the ratio of tidal volume to functional residual capacity) on aerated lung tissue [11]. Evidence supports the efficacy, in reducing ventilator-induced lung injury and mortality, of restraining tidal volume to 4–8 mL/kg of predicted body weight, maintaining plateau pressure below 30 cmH_2_O [12], maintaining driving pressure (the difference between plateau pressure and positive end-expiratory pressure) below 13 cmH_2_O [13], and reducing respiratory rate [14]. These approaches are grounded in fundamental thermodynamic principles, suggesting that lung injury arises from the transfer of mechanical power from the ventilator to the patient, leading to energy dissipation within the lungs, potentially resulting in heat generation, inflammation, and structural deformation of cells and the extracellular matrix [15].

**Figure 1:**
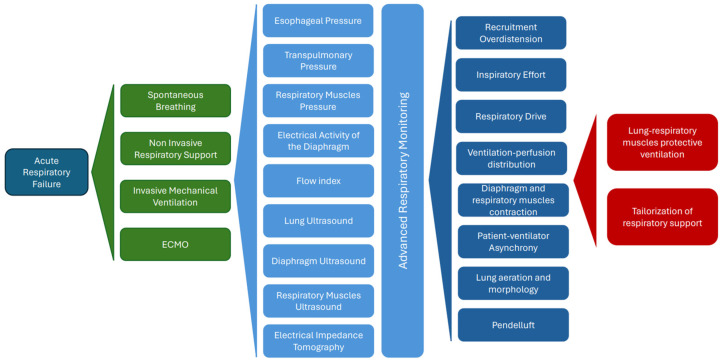
Patients with acute respiratory failure necessitate different ventilatory supports (green boxes) according to disease severity. In these patients, several advanced respiratory monitoring techniques (light blue boxes) can be applied to assess and resolve several important issues (dark blue boxes). This approach aims to guarantee protective mechanical ventilation to the lung and respiratory muscles, tailored to the characteristics of every single patient (red boxes).

Advanced respiratory monitoring holds the potential to play a pivotal role in assessing lung morphology and further optimizing mechanical ventilation to safeguard both lung parenchyma and respiratory muscles. Consequently, it promises to facilitate the early identification of patients at increased risk of lung and respiratory muscle injuries [16] (Figure 1).

**Figure 2:**
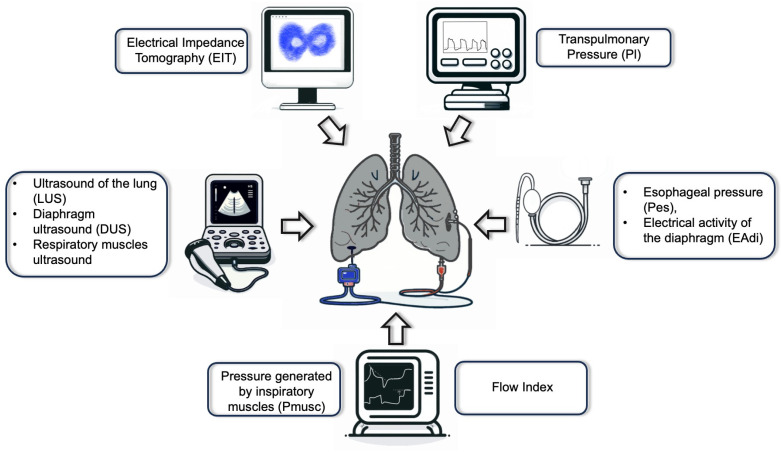
In patients with acute respiratory failure, even those receiving extracorporeal membrane oxygenation, advanced respiratory monitoring must be used including different tools like electrical impedance tomography, esophageal and transpulmonary pressures, lung and diaphragm ultrasonography, the electrical activity of the diaphragm, and flow index.

The present review is focused on describing bedside tools employed for advanced respiratory monitoring, i.e., the assessment of transpulmonary pressure (Pl), esophageal pressure (Pes), pressure generated by inspiratory muscles (Pmusc), flow index, and electrical activity of the diaphragm (EAdi), as well as ultrasound of the lung (LUS), diaphragm (DUS), and respiratory muscles, and electrical impedance tomography (EIT), with a particular emphasis on patients undergoing ECMO (Figure 2).

## 2. Lung Recruitment and Overdistention: Esophageal Pressure and Transpulmonary Pressure

Esophageal pressure (Pes) is assessed through a dedicated esophageal catheter equipped with a balloon [17] to estimate pleural pressure (Ppl) [18].

Proper placement of the esophageal balloon is essential, with it typically situated approximately 35–40 cm from the nostril, beyond the heart, and inflated with air.

**Figure 3:**
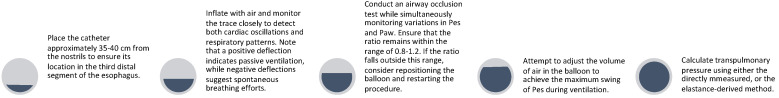
The steps required to achieve correct esophageal catheter positioning are summarized from left to right. In order to obtain reliable measures, all of the steps need to be strictly followed.

Transpulmonary pressure (Pl) denotes the pressure disparity between the alveoli and the pleural space. This is determined by measuring the difference between alveolar pressure and Ppl [17]. When assessed without airflow, Pl signifies the pressure acting solely on the lung, assuming open airways [17]. Ensuring the reliability of Pes measurements is paramount, with this requiring adherence to a series of meticulous steps [19]. Correct positioning is verified by observing changes in the tracing pattern, indicative of passive or spontaneously breathing patients. Additionally, cardiac oscillations can help confirm proper positioning. An occlusion test is necessary to rule out mispositioning, with the Pes-to-airway pressure ratio ideally falling within the range of 0.8–1.2 [20]. Ultimately, Pes evaluation can be influenced by various effects [21], reflecting the elastic properties of the esophageal balloon and the repercussions determined on the esophageal wall. Calibration procedures typically mitigate these artifacts. This involves adjusting the volume of air insufflated into the esophageal balloon to achieve the maximum change in the trace between end-inspiration and expiration [22,23]. These factors underscore the importance of experience in using Pes measurements, as well as ongoing debates regarding their reliability in estimating Ppl. Figure 3 summarizes the steps required to obtain reliable values from esophageal catheter measurements.

During mechanical ventilation, the pressure delivered to the respiratory system facilitates the expansion of the chest wall and lungs, overcoming resistive loads in the presence of airflow [17]. The monitoring and analysis of the generated pressures are of clinical relevance, aiding in discriminating the contribution made by the lung and chest wall.

In supine patients, Ppl follows a ventral-to-dorsal gradient, resulting in higher Ppl in dependent lung regions compared to non-dependent zones. This sternal-to-vertebral Ppl gradient directly corresponds to the vertical superimposed pressure gradient across the lung [24,25]. A vertical pressure gradient has been observed in both healthy individuals and those with ARDS [26]. In ARDS, the superimposed pressure gradient is elevated due to disrupted gas-to-tissue ratio distribution throughout the lung [26]. In experimental research involving lung-injured pigs with direct monitoring of Ppl and Pes, expiratory Pl computed from Pes aligned with expiratory distending pressure in dependent and mid-lung regions at varying positive end-expiratory pressures (PEEP) [24]. In the same setting, inspiratory distending pressure correlated with inspiratory Pl computed through elastance-derived methods in non-dependent lung regions and inspiratory Pes-computed Pl in mid and dependent lung regions [24].

The mechanical properties of the lung and chest may be compromised by different conditions during ARDS [27]. Lung impairment typically results from the underlying disease, while chest wall abnormalities can stem from factors like edema, abdominal distension, and pleural effusion occurring during IMV [27]. In ARDS patients undergoing IMV, Pl assessment allows for the guiding of lung recruitment and the avoidance of overdistention. Setting PEEP to counter expiratory Pl has been suggested in ARDS patients and healthy subjects undergoing elective pelvic robotic surgery [28,29,30]. Recently, the EPVent2 trial has demonstrated that a Pes-guided strategy resulted in no significant difference in death and days free from mechanical ventilation when compared to a high-PEEP/FiO_2_ table in patients intubated for moderate-to-severe ARDS [29].

In a cohort of patients diagnosed with influenza A (H1N1)-related ARDS who were referred to a regional ECMO center due to refractory hypoxemia under IMV, the implementation of PEEP guided by Pl yielded several benefits. It should be noted, however, that this approach assumed that the measurement of Pes remained unchanged in relation to changes in lung volume or local gradients in pleural pressure. This assumption may have led to imprecise conclusions. Nevertheless, this approach resulted in the improved mechanical characteristics of the respiratory system, enhanced oxygenation, and facilitated the implementation of a protective lung ventilation strategy [31]. Moreover, the partitioning of respiratory mechanics between the lung and chest wall proved crucial in discerning whether the applied ventilator pressure was directed toward the lung or dissipated across a stiff chest wall. This approach helps to identify which patients could benefit from vv-ECMO [31]. Following this approach based on the open lung concept guided by Pl, patients who fulfilled all of the criteria for vv-ECMO, as outlined in the EOLIA trial [4], showed an increase in oxygenation and improved lung respiratory compliance. Consequently, it can prevent the need for ECMO [32]. PEEP can also be set according to the lowest elastance of the respiratory system identified during a stepwise decreasing PEEP trial [33]. However, the lowest elastance of the respiratory system might be associated with negative end-expiratory Pl [34].

On the other hand, ventilator-induced lung injury (VILI) is sustained by regional overdistention occurring during protective tidal ventilation [35]. Both inspiratory Pl and transpulmonary driving pressure reflect the direct pressure exerted on the lung during insufflation. Limiting inspiratory Pl derived from elastance to 20–25 cmH_2_O appears reasonable to prevent overdistention in non-dependent lung regions [36]. Confirming previous results [29], a transpulmonary driving pressure ≥12 cmH_2_O, rather than an elastance-derived inspiratory Pl ≥ 24 cmH_2_O, showed as a mortality risk factor at 60 days.

In ARDS patients undergoing vv-ECMO, a ventilator strategy based on Pl helped clinicians to identify a higher PEEP, ensuring lower driving pressure and mechanical power, when compared to a lung rest ventilator strategy [37]. This Pl-based approach improved weaning success from vv-ECMO (from 48% to 71%) and reduced the levels of some cytokines (i.e., IL-1β, IL-6, and IL-8) [37]. In addition, mortality at 6 months was also reduced from 56% to 36% [37].

It is recognized that the use of an ultraprotective ventilatory strategy that combines lower tidal volume, lower plateau pressure, and lower driving pressure is associated with a reduced inflammatory response [38]. Therefore, applying advanced respiratory monitoring to identify optimal mechanical ventilation strategies and settings could facilitate a reduction in the risk of VILI and improve patient outcomes.

Since the goal of vv-ECMO is to guarantee oxygen delivery to organs while minimizing VILI [39,40,41], apneic oxygenation may also be a viable option to reduce the mechanical power applied to the lungs [33]. Once lung dysfunction resolves, IMV and spontaneous breathing can be progressively reinstated to allow for vv-ECMO weaning [39] and prevent diaphragm dysfunction [42].

Spontaneous breathing offers several advantages for the lung and diaphragm in ARDS patients, enhancing gas exchange and preventing muscular atrophy [43,44,45]. However, in severe ARDS, both spontaneous and assisted breathing may have detrimental effects on the injured lung and diaphragm. In spontaneously breathing patients and those receiving properly configured NIRS or assisted IMV for severe ARDS, forceful inspiratory efforts may cause an excessive drop in Ppl and an increase in dynamic and static alveolar pressure, non-uniformly distributed throughout the lungs [46]. In these scenarios, dependent lung regions close to the diaphragm undergo more pronounced Ppl fluctuations compared to aerated non-dependent lung regions. This leads to the *pendelluft* phenomenon (i.e., alveolar gas switches from non-dependent to dependent lung zones at the start of inspiration), a recognized risk factor for patient self-induced lung injury (P-SILI) [46]. For patients admitted with severe ARDS at risk of P-SILI, despite the absence of definitive Pl reference values, maintaining Pl below the upper limit of 20–25 cmH_2_O should help to mitigate harmful inspiratory efforts during active or assisted breath [36].

A strategy for lung- and diaphragm-protective ventilation has also been attempted in vv-ECMO patients through the modulation of sedation, ventilator settings, and sweep gas flow [47]. Increasing sweep gas flow effectively and consistently reduced respiratory effort and lung-distending pressure [47]. The increase in sweep gas flow was shown to reduce the swing of Pes not only in intubated ARDS patients [47] but also in patients undergoing vv-ECMO during spontaneous breathing or NIRS because of COPD exacerbation, ARDS, or as a bridge to lung transplantation [48]. Therefore, Pes monitoring would be advisable to guarantee safety and to guide clinicians in ventilator and ECMO settings.

Pes can also guide weaning from vv-ECMO in patients with spontaneous breathing activity during a stepwise decrease in sweep gas flow. In fact, if negative swings of Pes are <15 cm H_2_O at a respiratory rate of <30 breaths/min, this can facilitate the safe decannulation of the patient [49].

In conclusion, the positioning of an esophageal catheter enables the assessment of Pes and Pl. These pressures serve as valuable guides for clinicians in determining the optimal PEEP for each patient, ensuring positive Pl at expiration. Furthermore, Pl can assist ICU physicians in establishing a protective tidal volume, with the aim of limiting inspiratory Pl to 20–25 cmH_2_O. It is important to note that utilizing an esophageal catheter demands trained and skilled personnel for its proper positioning, calibration, and measurement assessments. For these reasons, the use of Pes and Pl is not generally employed in clinical practice but instead only by a few expert centers or in research settings.

## 3. Lung Aeration: Lung Ultrasound and Electrical Impedance Tomography

ARDS is characterized by several physio-pathological modifications of the lungs whereby consolidation due to inflammatory edema of alveolar and interstitial spaces, congestion of pulmonary capillaries, and atelectasis alternate with normally aerated areas [50]. In ARDS patients, a computed tomography (CT) scan is the standard radiological examination to evaluate lung morphology and assess the aeration changes resulting from PEEP and prone positioning application [50]. However, the use of radiation and its non-applicability at the bedside limit CT scan execution. LUS and EIT may represent valid tools to constantly assess lung aeration in ARDS patients at the bedside.

LUS evaluates lung morphology in real time [51]. As previously described [51,52], different patterns and scores of lung aeration can be assessed through LUS by switching from a normally aerated lung to consolidation: normal aeration (A-pattern—score 0), characterized by the reverberation of a sliding pleural line at regular intervals (lines A), eventually associated with B-lines < 3; moderate loss of aeration (B1-pattern—score 1) with well-spared B-lines ≥ 3 at regular interval or coalescent B lines originating from <50% of the pleural line; and severe aeration loss (B2-pattern—score 2) with multiple coalescent B-lines originating from >50% of the pleural line (total loss of aeration (C-pattern—score 3) with consolidation expressed by a tissue-like pattern.

In case of complete loss of aeration, LUS permits its characterization into inflammatory consolidation or atelectasis based on the presence or absence of a dynamic bronchogram, respectively, with a close correlation with CT scans [53].

The thorax can be scanned over twelve zones through LUS, six per hemithorax, delineated by the sternum, anterior, and posterior axillary lines in the anterior, lateral, and posterior regions, with each one divided into superior and inferior areas. Thus, the global LUS score ranges from a minimum of 0 (best aeration) to a maximum of 36 (total loss of aeration) [51]. LUS application in critically ill patients has progressively grown in the last 30 years [54]. In ARDS patients, lung monitoring using LUS has been demonstrated to be useful for tracking disease progression over time and evaluating the response to therapies [55,56]. In particular, LUS allows for the assessment of lung recruitment following PEEP application [55] as well as the improvement of fluid administration during ARF [56]. LUS helps in the identification of patients potentially responsive to prone positioning according to the focal distribution of the disease [57]. Moreover, LUS has been demonstrated to be useful in identifying patients at risk of weaning failure due to weaning-induced pulmonary edema [58]. From this perspective, a comprehensive ultrasound assessment involving the lung, heart, and respiratory muscles is desirable to support clinical judgement in dealing with patients undergoing weaning from IMV [59,60,61]. LUS has been demonstrated to correlate with findings in CT scans or chest X-rays, even in patients undergoing ECMO [62]. For this reason, serial evaluations with LUS were extensively used during the recent COVID-19 pandemic in ECMO patients instead of CT scans or chest X-rays [63,64]. LUS can be used daily to monitor the modification of lung consolidation [65] and for the early detection of the development of hospital-acquired pneumonia [66]. Typical features have been reported to be the consolidation of lower lobes, diffuse pulmonary edema, and the presence of a color Doppler intrapulmonary flow or dynamic air bronchogram within consolidations [66]. LUS score has a strong negative association with the severity of lung disease, as assessed through the dynamic compliance of the respiratory system [67]. LUS can also quantify lung recruitment in ARDS patients undergoing ECMO [68]; this is of clinical interest since patients with a high potential for lung recruitment have a shorter ICU stay and ECMO duration [69]. In addition, the maximum LUS value is highly predictive of the prognosis of COVID-19 patients [70]. Ultimately, LUS offers the benefit of decreasing healthcare expenses for patients with ARDS, and it does not require the transport of patients to radiological departments for chest CT scans, thereby reducing the associated risks [71].

Besides these advantages, LUS does not allow for the assessment of overdistension during mechanical ventilation [50]. In addition, despite its extensive use during the COVID-19 pandemic both in and outside the ICU to provide a qualitative and quantitative description of lung involvement [51], there is a learning curve; at least 25 supervised examinations seem to be necessary to achieve basic competency in LUS. Furthermore, no consensus exists on the advanced/quantitative assessment of lung aeration [72].

EIT is a noninvasive, radiation-free, bedside, and real-time lung monitoring technique that tracks the modification of lung ventilation. EIT examination consists of the placement of a silicon belt with 16 to 32 electrodes, between the fourth and sixth intercostal space. By applying low currents through pairs of electrodes, EIT measures the resulting voltages from impedance changes, and it provides data on lung ventilation and perfusion [73,74]. Through the assessment of ventilation distribution and end-expiratory lung volume, EIT is a valuable tool to assist clinicians in defining the optimal personalized ventilator settings (i.e., PEEP and tidal volume) in patients with ARF and/or ARDS [73,75].

In ARDS patients, the application of high PEEP may be detrimental and associated with alveolar overdistension and hemodynamic instability [76]. EIT can assess the recruited and overdistended lung volume at changes in PEEP values by analyzing the variation in end-expiratory lung impedance (EELI) [77]. The “optimal” PEEP value can be easily detected by performing a decremental PEEP trial [78]. During a decremental PEEP trial after a maximal recruiting maneuver, the “optimal” PEEP value was defined by the intercept point of cumulated collapse and overdistension percentage curves [78]. This strategy has also been applied in patients receiving ECMO for different reasons [79,80,81,82], even after prone positioning during ECMO [83]. Something that is noteworthy is that the “optimal” PEEP selected with EIT was shown to have good concordance with the value selected by the physician according to the combination of mechanical respiratory criteria, cardiac ultrasonography, and/or hemodynamic tolerance [80,81].

Another method, described below, was proposed by Eronia et al. [77]. According to this procedure [77], after the application of a recruiting maneuver, a PEEP value was set. If the EELI decreased more than 10% within 10 min after recruitment, PEEP was increased by 2 cmH_2_O, and recruitment was reapplied. In turn, the “optimal” PEEP value was defined as the lowest one avoiding an EELI decrease of <10% [77]. As proposed by Zhao et al., the optimal PEEP is also supposed to be associated with more homogeneous gas distribution within the lung, i.e., the lowest inhomogeneity index [84].

EIT has been employed to assess alveolar gas distribution and *pendelluft* phenomenon in patients subjected to assisted IMV for ARF [85] and in an experimental acute lung injury model of spontaneous breathing [45]. It has been demonstrated that occult *pendelluft* increasingly occurs with the progressive reduction in ventilatory support [85] and increased spontaneous breathing effort [45]. Thus, in the presence of this anomalous alveolar gas distribution detected through EIT systems at the bedside, clinicians are facilitated in the timely application of all those corrective measures aimed at abolishing vigorous inspiratory effort and *pendelluft* and, consequently, preventing P-SILI [46].

EIT has been recently used to monitor lung ventilation/perfusion distribution mismatch too. Perfusion EIT is based on the administration of a 10 mL hypertonic (5 to 10%) saline bolus during an expiratory hold maneuver. This technique could be an adjunctive bedside tool to identify patients with pulmonary embolism [86] or to assess the modification of ventilation/perfusion mismatch after PEEP changes [87] or prone positioning [88], even in patients undergoing ECMO [89].

In summary, LUS and EIT are valuable bedside imaging techniques, each contributing unique data. LUS aids in evaluating lung edema and consolidation, while EIT focuses on ventilation distribution and overdistension detection. These combined insights assist clinicians in tailoring individualized PEEP settings for ARDS patients, including those on vv-ECMO support. Despite requiring some training, their noninvasive nature has led to the widespread adoption of these advanced monitoring tools.

## 4. Inspiratory Effort: Pressure Generated by Inspiratory Muscles, Flow Index, Electrical Activity of the Diaphragm, and Ultrasound of Respiratory Muscles

During spontaneous breathing, inspiratory muscles generate pressure (Pmus), defined as the difference between chest wall recoil (Pcw) and Pes swing [36]. The pressure–time product of Pes (PTPes), i.e., the integral of Pes variation over inspiratory time, represents the parameter used to assess the patient’s inspiratory effort [17].

In four COPD patients failing to wean from IMV and set to undergo extracorporeal carbon dioxide removal (ECCO_2_-R), PTPes were also used to measure inspiratory effort before and after ECCO_2_-R during a T-piece trial [90]. In this report, the authors demonstrated that decreasing inspiratory effort was likely to cause a decrease in the CO_2_ production of respiratory muscles. In patients with hypercapnia, this reduction contributes to an overall decrease in total CO_2_ production. In such scenarios, the use of ECCO_2_-R may effectively remove enough CO_2_ to lower ventilator demand, potentially enabling spontaneous unassisted breathing [90].

More recently, direct interpretation of flow waveform showed a strong correlation with Pes-derived data [91,92]. Flow index mathematically describes the pattern of the flow waveform during inspiratory phases [92]. The elevated flow index defines vigorous inspiratory effort and closely relates to elevation in Pmus and PTPes values. Cut-off values have also been defined: a flow index higher than 4.5 accurately detects breaths with high inspiratory effort, whereas a flow index lower than 2.6 predicts low inspiratory effort [91].

Electrical activity of the diaphragm (EAdi) is the signal closest to respiratory centers’ output, measurable at the bedside [93]. Similar to Pes, EAdi monitoring requires invasive monitoring, but it allows for the driving of neurally adjusted ventilatory assist [94]. EAdi varies according to the patient’s level of assistance, and its peak values tightly correlate with Pes and Pmus to quantify inspiratory effort [95]. In this regard, the ratio between the pressure generated against an occluded airway (during expiratory hold) and the corresponding signal from EAdi is defined as neuro-mechanical efficiency of the diaphragm, and it strongly correlates with the information conveyed by Pes during tidal ventilation [96].

EAdi monitoring has proven feasible in patients undergoing vv-ECMO while recovering from ARDS [97,98]. Indeed, the increase in sweep gas flow allows for an increase in carbon dioxide clearance from the membrane lung (i.e., oxygenator), while reducing breathing effort [97,98]. All in all, EAdi provides useful clinical information and can efficiently guide protective assisted ventilation [96,99]. Nonetheless, diaphragm weakness and activation of accessory inspiratory muscles are both common in critical illness. This may contribute to the underestimation of the patient’s real effort with EAdi. Furthermore, wide heterogeneity in the EAdi signal has been documented [99], prompting further research on its meaning.

Assessing respiratory muscles through ultrasound provides a noninvasive, reproducible tool readily available at the bedside. Diaphragm ultrasonography (DUS) requires quick training [100]; however, it does not permit continuous monitoring. During the respiratory cycle, DUS can quantitatively estimate variation in diaphragm thickness and thickening fraction, providing an estimate of inspiratory effort [101]. Diaphragmatic thickening fraction has been shown to have a tight correlation with PTPes and EAdi [101]. In patients undergoing veno-arterial ECMO, thickening fraction was used to monitor the modification of inspiratory effort at varying sweep gas flows [102], with the findings being in line with those of other studies [97,98]. Clinically relevant outcomes have recently been linked to variation in diaphragmatic thickening fraction [43]. Both increasing and decreasing diaphragmatic thickening fraction are related to prolonged ventilation and poor outcomes [43,103]; this is possibly due to diaphragm injury from under- or over-assistance [43]. Apparently, improved outcomes and the shortest duration of ventilation are related to maintaining a diaphragmatic thickening fraction close to 15–30% [43]. Thickening fraction was also used to identify the presence of diaphragm dysfunction in ARDS patients undergoing veno-venous ECMO [104]. In this population, the presence of diaphragm dysfunction after 7 days of ECMO was not associated with the cumulative percentage of spontaneous breathing or any other clinical outcomes [104]. Another parameter assessed through DUS is diaphragmatic excursion during spontaneous breathing [105,106]. Its combination with the rapid shallow breathing index during a T-piece trial enhanced the prediction of weaning failure [105,107]. A recent consensus highlighted other ultrasound techniques as possible advances in diaphragm evaluation [105]. Recent trials on both healthy subjects and critically ill patients showed tissue Doppler imaging of diaphragmatic excursion as a valid technique to track diaphragm excursion kinetics [108,109]. Diaphragmatic excursion tissue Doppler imaging-derived parameters showed significant differences between weaning success and failure [109] as well as extubation success and failure [108]. Namely, weaning failure and extubation failure were strongly associated with higher peak contraction velocities and more rapid diaphragmatic relaxation [108,109]. Possibly, these represent dynamic compensations for excessive muscular load [108,109]. Diaphragmatic speckle tracking is another fascinating ultrasound evaluation of tissue contraction [110]. New diaphragmatic speckle tracking algorithms have been recently evaluated in healthy diaphragm assessment, detecting increased diaphragmatic effort with high reliability [110].

Finally, sonographic assessment of expiratory muscles has recently been proven possible in mechanically ventilated patients [111]. Integrated assessment of critically ill patient respiratory dynamics represents a challenge for further research.

In conclusion, assessing inspiratory effort and muscles involves multiple techniques that complement each other in understanding the complex axis from respiratory centers (EAdi) to muscular contraction (Pes and DUS). While EAdi and Pes may not be universally available at all centers, DUS offers significant advantages. It is easy to learn and perform, requiring only a standard ultrasonographic machine, which is typically available in all ICUs.

## 5. Patient–Ventilator Asynchrony: Esophageal Pressure, Electrical Activity of the Diaphragm, and Diaphragm Ultrasound

Patient–ventilator asynchrony, defined as a lack of coordination between patient effort and ventilator assistance during both IMV and NIRS [112,113], poses a significant challenge in patient care. Incidences of asynchronous events exceeding 10% occur in up to 25% of patients undergoing IMV and in up to 80% of those receiving NIRS [112,113]. Importantly, high incidence has been associated with worsened patient outcomes [112]. Recognizing patient–ventilator asynchrony is crucial for implementing corrective maneuvers to reduce its occurrence [112].

Although initially proposed, the observation of ventilator waveforms has low sensitivity in identifying asynchronous events for both expert and non-expert physicians, during both IMV and NIRS, indicating the need for additional signals, such as Pes [17] and EAdi [112,113]. DUS has recently been proposed to aid physicians in recognizing asynchronous events during IMV [114] and NIRS [115] at the bedside. However, this method, while highly performant, requires the import of ventilator waveforms into the ultrasound machine during diaphragm displacement assessment [115].

Some automatic and validated algorithms have been proposed and validated to recognize patient–ventilator asynchrony, through the analysis of the entire ventilator waveform [116] or solely the expiratory flow curve [117]. Other proposed algorithms for the detection of asynchronies are based on airflow spectral analysis [118] or the equation of motion [119]. Finally, a computerized method combining EAdi with flow and Paw signal analysis assures greater accuracy of asynchronous detection as compared to visual inspection of ventilator curves [120].

In ARDS patients receiving vv-ECMO, a few are known to display patient–ventilator asynchrony. In 10 ARDS patients undergoing vv-ECMO, Mauri et al. reported a high incidence of asynchronous events during PSV with two different cycling-off settings; of note, the incidence of asynchronies was inversely correlated with respiratory system static compliance [121].

Patient–ventilator interaction remains a significant concern during assisted mechanical ventilation, even in patients supported by vv-ECMO. These interactions have been associated with adverse outcomes, underscoring the importance of monitoring them closely with appropriate signals. Implementing corrective measures to minimize their occurrence is crucial for optimizing patient care.

## 6. Advanced Respiratory Monitoring in Weaning from vv-ECMO

Before considering weaning from vv-ECMO and preparing for decannulation, it is crucial to assess the adequacy of gas exchange reserve. Specifically, it must be guaranteed that there is a PaO_2_ ≥ 70 mmHg with an FiO_2_ ≤ 60% and a PEEP ≤ 10 cmH_2_O. Additionally, the mechanical ventilator settings should include a tidal volume of 6 mL/kg of predicted body weight, with a plateau pressure ≤ 28 cmH_2_O and a respiratory rate ≤ 28 breaths/min. For the initial assessment of oxygenation capability, one approach is to decrease ECMO flow to 1–1.5 LPM while ensuring the patient maintains adequate oxygenation. Alternatively, ECMO flow can be maintained while gradually weaning the fraction of delivered oxygen. To evaluate ventilatory reserve, the patient should tolerate a low sweep gas flow (<2 LPM). These conditions should result in acceptable pH and PaCO_2_ levels without excessive work for breathing and/or an excessive respiratory rate. As weaning is a gradual process spanning hours to days, arterial blood gases should be monitored regularly throughout the entire process [39].

In the vv-ECMO weaning process, advanced respiratory monitoring may be of guidance. For example, the assessment of Pes and Pl may guide clinicians toward settings with the best PEEP, avoiding overdistension [29]. This may also be guided using EIT [79,80,81,82]. As already mentioned above, Pes can also serve as a valuable guide for weaning from vv-ECMO in patients exhibiting spontaneous breathing activity during a stepwise decrease in sweep gas flow. Specifically, if negative swings of Pes are <15 cmH_2_O at a respiratory rate of <30 breaths/min, this can facilitate the safe decannulation of the patient [49]. Another important issue to be investigated and detected during vv-ECMO weaning is the presence of diaphragm dysfunction, which can be assessed through DUS. Although not associated with the worsening of vv-ECMO outcomes [104], it is well known that the presence of diaphragm dysfunction prolongs weaning from mechanical ventilation and may predict weaning failure [105,107].

## 7. Conclusions

Regarding the optimal treatment of ARDS, there are still many aspects that need clarification. These encompass a spectrum of issues, including the optimization of spontaneous breathing, the adjustment of tidal volume, and the customization of ventilation parameters based on lung volume and perfusion. Furthermore, there is ongoing debate regarding the effectiveness of higher PEEP compared to prone positioning, as well as the preference for alternative ventilation modes such as airway pressure release ventilation (APRV) and high-frequency oscillatory ventilation (HFOV). Additionally, the potential expansion of extracorporeal carbon dioxide removal and vv-ECMO applications warrants further investigation [122,123], which will undoubtedly be the focus of future investigations. Against this backdrop, ensuring adequate monitoring, particularly in complex cases requiring extracorporeal support, becomes paramount. Such vigilant monitoring not only yields crucial insights into lung status but also facilitates the optimization and personalization of therapies, with the ultimate goal of enhancing patient outcomes. So far, there has not been a definitive superiority of one technique over another, particularly in the context of patients undergoing vv-ECMO. Consequently, each center should choose its techniques based on experience, clinical purpose, and, last but not least, personalizing the choice based on the patient and the disease’s severity. This aspect is of pivotal importance in the perspective of tailoring mechanical ventilation to the patient rather than the disease.

## Data Availability

Not applicable.

## References

[1] Bellani G., Laffey J.G., Pham T., Fan E., Brochard L., Esteban A., Gattinoni L., van Haren F., Larsson A., McAuley D.F. (2016). Epidemiology, patterns of care, and mortality for patients with acute respiratory distress syndrome in intensive care units in 50 countries. JAMA.

[2] Grotberg J.C., Reynolds D., Kraft B.D. (2023). Management of severe acute respiratory distress syndrome: A primer. Crit. Care.

[3] Goligher E.C., Tomlinson G., Hajage D., Wijeysundera D.N., Fan E., Juni P., Brodie D., Slutsky A.S., Combes A. (2018). Extracorporeal membrane oxygenation for severe acute respiratory distress syndrome and posterior probability of mortality benefit in a post hoc bayesian analysis of a randomized clinical trial. JAMA.

[4] Combes A., Hajage D., Capellier G., Demoule A., Lavoue S., Guervilly C., Da Silva D., Zafrani L., Tirot P., Veber B. (2018). Extracorporeal membrane oxygenation for severe acute respiratory distress syndrome. N. Engl. J. Med..

[5] Pham T., Combes A., Roze H., Chevret S., Mercat A., Roch A., Mourvillier B., Ara-Somohano C., Bastien O., Zogheib E. (2013). Extracorporeal membrane oxygenation for pandemic influenza a(H1N1)-induced acute respiratory distress syndrome: A cohort study and propensity-matched analysis. Am. J. Respir. Crit. Care Med..

[6] Noah M.A., Peek G.J., Finney S.J., Griffiths M.J., Harrison D.A., Grieve R., Sadique M.Z., Sekhon J.S., McAuley D.F., Firmin R.K. (2011). Referral to an extracorporeal membrane oxygenation center and mortality among patients with severe 2009 influenza a(h1n1). JAMA.

[7] Peek G.J., Mugford M., Tiruvoipati R., Wilson A., Allen E., Thalanany M.M., Hibbert C.L., Truesdale A., Clemens F., Cooper N. (2009). Efficacy and economic assessment of conventional ventilatory support versus extracorporeal membrane oxygenation for severe adult respiratory failure (CESAR): A multicentre randomised controlled trial. Lancet.

[8] Bertoni M., Spadaro S., Goligher E.C. (2020). Monitoring patient respiratory effort during mechanical ventilation: Lung and diaphragm-protective ventilation. Crit. Care.

[9] Brochard L., Slutsky A., Pesenti A. (2017). Mechanical ventilation to minimize progression of lung injury in acute respiratory failure. Am. J. Respir. Crit. Care Med..

[10] Tonetti T., Vasques F., Rapetti F., Maiolo G., Collino F., Romitti F., Camporota L., Cressoni M., Cadringher P., Quintel M. (2017). Driving pressure and mechanical power: New targets for VILI prevention. Ann. Transl. Med..

[11] Gattinoni L., Carlesso E., Caironi P. (2012). Stress and strain within the lung. Curr. Opin. Crit. Care.

[12] Brower R.G., Matthay M.A., Morris A., Schoenfeld D., Thompson B.T., Wheeler A. (2000). Ventilation with lower tidal volumes as compared with traditional tidal volumes for acute lung injury and the acute respiratory distress syndrome. N. Engl. J. Med..

[13] Amato M.B., Meade M.O., Slutsky A.S., Brochard L., Costa E.L., Schoenfeld D.A., Stewart T.E., Briel M., Talmor D., Mercat A. (2015). Driving pressure and survival in the acute respiratory distress syndrome. N. Engl. J. Med..

[14] Hotchkiss J.R., Blanch L., Murias G., Adams A.B., Olson D.A., Wangensteen O.D., Leo P.H., Marini J.J. (2000). Effects of decreased respiratory frequency on ventilator-induced lung injury. Am. J. Respir. Crit. Care Med..

[15] Cressoni M., Gotti M., Chiurazzi C., Massari D., Algieri I., Amini M., Cammaroto A., Brioni M., Montaruli C., Nikolla K. (2016). Mechanical power and development of ventilator-induced lung injury. Anesthesiology.

[16] Cammarota G., Simonte R., Longhini F., Spadaro S., Vetrugno L., De Robertis E. (2023). Advanced point-of-care bedside monitoring for acute respiratory failure. Anesthesiology.

[17] Akoumianaki E., Maggiore S.M., Valenza F., Bellani G., Jubran A., Loring S.H., Pelosi P., Talmor D., Grasso S., Chiumello D. (2014). The application of esophageal pressure measurement in patients with respiratory failure. Am. J. Respir. Crit. Care Med..

[18] Dornhorst A.C., Leathart G.L. (1952). A method of assessing the mechanical properties of lungs and air-passages. Lancet.

[19] Jonkman A.H., Telias I., Spinelli E., Akoumianaki E., Piquilloud L. (2023). The oesophageal balloon for respiratory monitoring in ventilated patients: Updated clinical review and practical aspects. Eur. Respir. Rev..

[20] Piquilloud L., Beitler J.R., Beloncle F.M. (2024). Monitoring esophageal pressure. Intensive Care Med..

[21] Hedenstierna G., Jarnberg P.O., Torsell L., Gottlieb I. (1983). Esophageal elastance in anesthetized humans. J. Appl. Physiol. Respir. Environ. Exerc. Physiol..

[22] Cammarota G., Lauro G., Santangelo E., Sguazzotti I., Perucca R., Verdina F., Boniolo E., Tarquini R., Bignami E., Mongodi S. (2020). Mechanical ventilation guided by uncalibrated esophageal pressure may be potentially harmful. Anesthesiology.

[23] Mojoli F., Iotti G.A., Torriglia F., Pozzi M., Volta C.A., Bianzina S., Braschi A., Brochard L. (2016). In vivo calibration of esophageal pressure in the mechanically ventilated patient makes measurements reliable. Crit. Care.

[24] Yoshida T., Amato M.B.P., Grieco D.L., Chen L., Lima C.A.S., Roldan R., Morais C.C.A., Gomes S., Costa E.L.V., Cardoso P.F.G. (2018). Esophageal manometry and regional transpulmonary pressure in lung injury. Am. J. Respir. Crit. Care Med..

[25] Pelosi P., Goldner M., McKibben A., Adams A., Eccher G., Caironi P., Losappio S., Gattinoni L., Marini J.J. (2001). Recruitment and derecruitment during acute respiratory failure: An experimental study. Am. J. Respir. Crit. Care Med..

[26] Pelosi P., D’Andrea L., Vitale G., Pesenti A., Gattinoni L. (1994). Vertical gradient of regional lung inflation in adult respiratory distress syndrome. Am. J. Respir. Crit. Care Med..

[27] Pelosi P., Cereda M., Foti G., Giacomini M., Pesenti A. (1995). Alterations of lung and chest wall mechanics in patients with acute lung injury: Effects of positive end-expiratory pressure. Am. J. Respir. Crit. Care Med..

[28] Cammarota G., Lauro G., Sguazzotti I., Mariano I., Perucca R., Messina A., Zanoni M., Garofalo E., Bruni A., Della Corte F. (2020). Esophageal pressure versus gas exchange to set peep during intraoperative ventilation. Respir. Care.

[29] Beitler J.R., Sarge T., Banner-Goodspeed V.M., Gong M.N., Cook D., Novack V., Loring S.H., Talmor D. (2019). Effect of titrating positive end-expiratory pressure (peep) with an esophageal pressure-guided strategy vs. an empirical high peep-fio2 strategy on death and days free from mechanical ventilation among patients with acute respiratory distress syndrome: A randomized clinical trial. JAMA.

[30] Talmor D., Sarge T., O’Donnell C.R., Ritz R., Malhotra A., Lisbon A., Loring S.H. (2006). Esophageal and transpulmonary pressures in acute respiratory failure. Crit. Care Med..

[31] Grasso S., Terragni P., Birocco A., Urbino R., Del Sorbo L., Filippini C., Mascia L., Pesenti A., Zangrillo A., Gattinoni L. (2012). Ecmo criteria for influenza a (H1N1)-associated ards: Role of transpulmonary pressure. Intensive Care Med..

[32] van der Zee P., Dos Reis Miranda D., Meeder H., Endeman H., Gommers D. (2019). Vvecmo can be avoided by a transpulmonary pressure guided open lung concept in patients with severe ards. Crit. Care.

[33] Graf P.T., Boesing C., Brumm I., Biehler J., Muller K.W., Thiel M., Pelosi P., Rocco P.R.M., Luecke T., Krebs J. (2022). Ultraprotective versus apneic ventilation in acute respiratory distress syndrome patients with extracorporeal membrane oxygenation: A physiological study. J. Intensive Care.

[34] Krebs J., Pelosi P., Rocco P.R.M., Hagmann M., Luecke T. (2018). Positive end-expiratory pressure titrated according to respiratory system mechanics or to ardsnetwork table did not guarantee positive end-expiratory transpulmonary pressure in acute respiratory distress syndrome. J. Crit. Care.

[35] Protti A., Maraffi T., Milesi M., Votta E., Santini A., Pugni P., Andreis D.T., Nicosia F., Zannin E., Gatti S. (2016). Role of strain rate in the pathogenesis of ventilator-induced lung edema. Crit. Care Med..

[36] Mauri T., Yoshida T., Bellani G., Goligher E.C., Carteaux G., Rittayamai N., Mojoli F., Chiumello D., Piquilloud L., Grasso S. (2016). Esophageal and transpulmonary pressure in the clinical setting: Meaning, usefulness and perspectives. Intensive Care Med..

[37] Wang R., Sun B., Li X., Tang X., He H., Li Y., Yuan X., Li H., Chu H., Tong Z. (2020). Mechanical ventilation strategy guided by transpulmonary pressure in severe acute respiratory distress syndrome treated with venovenous extracorporeal membrane oxygenation. Crit. Care Med..

[38] Rozencwajg S., Guihot A., Franchineau G., Lescroat M., Brechot N., Hekimian G., Lebreton G., Autran B., Luyt C.E., Combes A. (2019). Ultra-protective ventilation reduces biotrauma in patients on venovenous extracorporeal membrane oxygenation for severe acute respiratory distress syndrome. Crit. Care Med..

[39] Tonna J.E., Abrams D., Brodie D., Greenwood J.C., Rubio Mateo-Sidron J.A., Usman A., Fan E. (2021). Management of adult patients supported with venovenous extracorporeal membrane oxygenation (VV ECMO): Guideline from the extracorporeal life support organization (ELSO). ASAIO J..

[40] Schmidt M., Pham T., Arcadipane A., Agerstrand C., Ohshimo S., Pellegrino V., Vuylsteke A., Guervilly C., McGuinness S., Pierard S. (2019). Mechanical ventilation management during extracorporeal membrane oxygenation for acute respiratory distress syndrome. An international multicenter prospective cohort. Am. J. Respir. Crit. Care Med..

[41] Marhong J.D., Telesnicki T., Munshi L., Del Sorbo L., Detsky M., Fan E. (2014). Mechanical ventilation during extracorporeal membrane oxygenation. An international survey. Ann. Am. Thorac. Soc..

[42] Goligher E.C., Dres M., Patel B.K., Sahetya S.K., Beitler J.R., Telias I., Yoshida T., Vaporidi K., Grieco D.L., Schepens T. (2020). Lung- and diaphragm-protective ventilation. Am. J. Respir. Crit. Care Med..

[43] Goligher E.C., Dres M., Fan E., Rubenfeld G.D., Scales D.C., Herridge M.S., Vorona S., Sklar M.C., Rittayamai N., Lanys A. (2018). Mechanical ventilation-induced diaphragm atrophy strongly impacts clinical outcomes. Am. J. Respir. Crit. Care Med..

[44] Goligher E.C., Fan E., Herridge M.S., Murray A., Vorona S., Brace D., Rittayamai N., Lanys A., Tomlinson G., Singh J.M. (2015). Evolution of diaphragm thickness during mechanical ventilation. Impact of inspiratory effort. Am. J. Respir. Crit. Care Med..

[45] Yoshida T., Uchiyama A., Matsuura N., Mashimo T., Fujino Y. (2013). The comparison of spontaneous breathing and muscle paralysis in two different severities of experimental lung injury. Crit. Care Med..

[46] Yoshida T., Uchiyama A., Fujino Y. (2015). The role of spontaneous effort during mechanical ventilation: Normal lung versus injured lung. J. Intensive Care.

[47] Dianti J., Fard S., Wong J., Chan T.C.Y., Del Sorbo L., Fan E., Amato M.B.P., Granton J., Burry L., Reid W.D. (2022). Strategies for lung- and diaphragm-protective ventilation in acute hypoxemic respiratory failure: A physiological trial. Crit. Care.

[48] Crotti S., Bottino N., Ruggeri G.M., Spinelli E., Tubiolo D., Lissoni A., Protti A., Gattinoni L. (2017). Spontaneous breathing during extracorporeal membrane oxygenation in acute respiratory failure. Anesthesiology.

[49] Al-Fares A.A., Ferguson N.D., Ma J., Cypel M., Keshavjee S., Fan E., Del Sorbo L. (2021). Achieving safe liberation during weaning from vv-ecmo in patients with severe ards: The role of tidal volume and inspiratory effort. Chest.

[50] Bitker L., Talmor D., Richard J.C. (2022). Imaging the acute respiratory distress syndrome: Past, present and future. Intensive Care Med..

[51] Mongodi S., De Luca D., Colombo A., Stella A., Santangelo E., Corradi F., Gargani L., Rovida S., Volpicelli G., Bouhemad B. (2021). Quantitative lung ultrasound: Technical aspects and clinical applications. Anesthesiology.

[52] Mongodi S., Bouhemad B., Orlando A., Stella A., Tavazzi G., Via G., Iotti G.A., Braschi A., Mojoli F. (2017). Modified lung ultrasound score for assessing and monitoring pulmonary aeration. Ultraschall Med..

[53] Lichtenstein D.A., Lascols N., Meziere G., Gepner A. (2004). Ultrasound diagnosis of alveolar consolidation in the critically ill. Intensive Care Med..

[54] Vetrugno L., Biasucci D.G., Deana C., Spadaro S., Lombardi F.A., Longhini F., Pisani L., Boero E., Cereser L., Cammarota G. (2024). Lung ultrasound and supine chest x-ray use in modern adult intensive care: Mapping 30 years of advancement (1993–2023). Ultrasound J..

[55] Chiumello D., Mongodi S., Algieri I., Vergani G.L., Orlando A., Via G., Crimella F., Cressoni M., Mojoli F. (2018). Assessment of lung aeration and recruitment by ct scan and ultrasound in acute respiratory distress syndrome patients. Crit. Care Med..

[56] Caltabeloti F., Monsel A., Arbelot C., Brisson H., Lu Q., Gu W.J., Zhou G.J., Auler J.O., Rouby J.J. (2014). Early fluid loading in acute respiratory distress syndrome with septic shock deteriorates lung aeration without impairing arterial oxygenation: A lung ultrasound observational study. Crit. Care.

[57] Haddam M., Zieleskiewicz L., Perbet S., Baldovini A., Guervilly C., Arbelot C., Noel A., Vigne C., Hammad E., Antonini F. (2016). Lung ultrasonography for assessment of oxygenation response to prone position ventilation in ards. Intensive Care Med..

[58] Ferre A., Guillot M., Lichtenstein D., Meziere G., Richard C., Teboul J.L., Monnet X. (2019). Lung ultrasound allows the diagnosis of weaning-induced pulmonary oedema. Intensive Care Med..

[59] Vetrugno L., Brussa A., Guadagnin G.M., Orso D., De Lorenzo F., Cammarota G., Santangelo E., Bove T. (2020). Mechanical ventilation weaning issues can be counted on the fingers of just one hand: Part 2. Ultrasound J..

[60] Vetrugno L., Guadagnin G.M., Brussa A., Orso D., Garofalo E., Bruni A., Longhini F., Bove T. (2020). Mechanical ventilation weaning issues can be counted on the fingers of just one hand: Part 1. Ultrasound J..

[61] Mayo P., Volpicelli G., Lerolle N., Schreiber A., Doelken P., Vieillard-Baron A. (2016). Ultrasonography evaluation during the weaning process: The heart, the diaphragm, the pleura and the lung. Intensive Care Med..

[62] Curry S., Tan A., Gargani L., Ng O., Roscoe A., Salaunkey K., Agrawal B., Vuylsteke A., Fowles J.A., Rubino A. (2021). Lung ultrasound and the role of lung aeration score in patients with acute respiratory distress syndrome on extracorporeal membrane oxygenation. Int. J. Artif. Organs.

[63] Taniguchi H., Ohta S., Honzawa H., Takahashi K., Iwashita M., Abe T., Takeuchi I. (2021). Usefulness of serial lung ultrasound for a severe COVID-19 patient on extracorporeal membrane oxygenation. Respir. Med. Case Rep..

[64] Moller-Sorensen H., Gjedsted J., Lind Jorgensen V., Lindskov Hansen K. (2020). COVID-19 assessment with bedside lung ultrasound in a population of intensive care patients treated with mechanical ventilation and ECMO. Diagnostics.

[65] Lu X., Arbelot C., Schreiber A., Langeron O., Monsel A., Lu Q. (2019). Ultrasound assessment of lung aeration in subjects supported by venovenous extracorporeal membrane oxygenation. Respir. Care.

[66] Pasqueron J., Dureau P., Arcile G., Duceau B., Hariri G., Lepere V., Lebreton G., Rouby J.J., Bougle A. (2022). Usefulness of lung ultrasound for early detection of hospital-acquired pneumonia in cardiac critically ill patients on venoarterial extracorporeal membrane oxygenation. Ann. Intensive Care.

[67] Ntoumenopoulos G., Buscher H., Scott S. (2021). Lung ultrasound score as an indicator of dynamic lung compliance during veno-venous extra-corporeal membrane oxygenation. Int. J. Artif. Organs.

[68] Dimopoulos S., Stefanidis K., Nanas S., Karabinis A. (2020). Quantifying lung recruitment and lung recovery in acute respiratory distress syndrome patients with venovenous extracorporeal membrane oxygenation support. Crit. Care Med..

[69] Camporota L., Caricola E.V., Bartolomeo N., Di Mussi R., Wyncoll D.L.A., Meadows C.I.S., Amado-Rodriguez L., Vasques F., Sanderson B., Glover G.W. (2019). Lung recruitability in severe acute respiratory distress syndrome requiring extracorporeal membrane oxygenation. Crit. Care Med..

[70] Schafer V.S., Recker F., Kretschmer E., Putensen C., Ehrentraut S.F., Staerk C., Fleckenstein T., Mayr A., Seibel A., Schewe J.C. (2023). Lung ultrasound in predicting outcomes in patients with COVID-19 treated with extracorporeal membrane oxygenation. Viruses.

[71] Cammarota G., Vetrugno L., Longhini F. (2023). Lung ultrasound monitoring: Impact on economics and outcomes. Curr. Opin. Anaesthesiol..

[72] Rouby J.J., Arbelot C., Gao Y., Zhang M., Lv J., An Y., Chunyao W., Bin D., Valente Barbas C.S., Dexheimer Neto F.L. (2018). Training for lung ultrasound score measurement in critically ill patients. Am. J. Respir. Crit. Care Med..

[73] Rauseo M., Spinelli E., Sella N., Slobod D., Spadaro S., Longhini F., Giarratano A., Gilda C., Mauri T., Navalesi P. (2022). Expert opinion document: “Electrical impedance tomography: Applications from the intensive care unit and beyond”. J. Anesth. Analg. Crit. Care.

[74] Longhini F., Maugeri J., Andreoni C., Ronco C., Bruni A., Garofalo E., Pelaia C., Cavicchi C., Pintaudi S., Navalesi P. (2019). Electrical impedance tomography during spontaneous breathing trials and after extubation in critically ill patients at high risk for extubation failure: A multicenter observational study. Ann. Intensive Care.

[75] Frerichs I., Amato M.B., van Kaam A.H., Tingay D.G., Zhao Z., Grychtol B., Bodenstein M., Gagnon H., Bohm S.H., Teschner E. (2017). Chest electrical impedance tomography examination, data analysis, terminology, clinical use and recommendations: Consensus statement of the translational eit development study group. Thorax.

[76] Mergoni M., Martelli A., Volpi A., Primavera S., Zuccoli P., Rossi A. (1997). Impact of positive end-expiratory pressure on chest wall and lung pressure-volume curve in acute respiratory failure. Am. J. Respir. Crit. Care Med..

[77] Eronia N., Mauri T., Maffezzini E., Gatti S., Bronco A., Alban L., Binda F., Sasso T., Marenghi C., Grasselli G. (2017). Bedside selection of positive end-expiratory pressure by electrical impedance tomography in hypoxemic patients: A feasibility study. Ann. Intensive Care.

[78] Costa E.L., Borges J.B., Melo A., Suarez-Sipmann F., Toufen C., Bohm S.H., Amato M.B. (2009). Bedside estimation of recruitable alveolar collapse and hyperdistension by electrical impedance tomography. Intensive Care Med..

[79] Di Pierro M., Giani M., Bronco A., Lembo F.M., Rona R., Bellani G., Foti G. (2022). Bedside selection of positive end expiratory pressure by electrical impedance tomography in patients undergoing Veno-Venous extracorporeal membrane oxygenation support: A comparison between COVID-19 ARDS and ARDS from other etiologies. J. Clin. Med..

[80] Soule C., Crognier L., Puel F., Ruiz S., Seguin T., Fourcade O., Georges B., Conil J.M., Minville V., Vardon-Bounes F. (2021). Assessment of electrical impedance tomography to set optimal positive end-expiratory pressure for venoarterial extracorporeal membrane oxygenation-treated patients. Crit. Care Med..

[81] Puel F., Crognier L., Soule C., Vardon-Bounes F., Ruiz S., Seguin T., Fourcade O., Minville V., Conil J.M., Georges B. (2020). Assessment of electrical impedance tomography to set optimal positive end-expiratory pressure for Veno-Venous ECMO-treated severe ards patients. J. Crit. Care.

[82] Franchineau G., Brechot N., Lebreton G., Hekimian G., Nieszkowska A., Trouillet J.L., Leprince P., Chastre J., Luyt C.E., Combes A. (2017). Bedside contribution of electrical impedance tomography to setting positive end-expiratory pressure for extracorporeal membrane oxygenation-treated patients with severe acute respiratory distress syndrome. Am. J. Respir. Crit. Care Med..

[83] Franchineau G., Brechot N., Hekimian G., Lebreton G., Bourcier S., Demondion P., Le Guennec L., Nieszkowska A., Luyt C.E., Combes A. (2020). Prone positioning monitored by electrical impedance tomography in patients with severe acute respiratory distress syndrome on veno-venous ecmo. Ann. Intensive Care.

[84] Zhao Z., Steinmann D., Frerichs I., Guttmann J., Moller K. (2010). Peep titration guided by ventilation homogeneity: A feasibility study using electrical impedance tomography. Crit. Care.

[85] Coppadoro A., Grassi A., Giovannoni C., Rabboni F., Eronia N., Bronco A., Foti G., Fumagalli R., Bellani G. (2020). Occurrence of pendelluft under pressure support ventilation in patients who failed a spontaneous breathing trial: An observational study. Ann. Intensive Care.

[86] Grassi L.G., Santiago R., Florio G., Berra L. (2020). Bedside evaluation of pulmonary embolism by electrical impedance tomography. Anesthesiology.

[87] Pavlovsky B., Pesenti A., Spinelli E., Scaramuzzo G., Marongiu I., Tagliabue P., Spadaro S., Grasselli G., Mercat A., Mauri T. (2022). Effects of peep on regional ventilation-perfusion mismatch in the acute respiratory distress syndrome. Crit. Care.

[88] Zarantonello F., Sella N., Pettenuzzo T., Andreatta G., Calore A., Dotto D., De Cassai A., Calabrese F., Boscolo A., Navalesi P. (2022). Early physiologic effects of prone positioning in COVID-19 acute respiratory distress syndrome. Anesthesiology.

[89] He H., Jiang J., Xu M., Yuan S., Long Y., Chi Y., Frerichs I., Zhao Z. (2022). Saline bolus-based electrical impedance tomography method for rapid bedside assessment of regional lung perfusion during ecmo therapy. Crit. Care.

[90] Pisani L., Fasano L., Corcione N., Comellini V., Guerrieri A., Ranieri M.V., Nava S. (2015). Effects of extracorporeal co2 removal on inspiratory effort and respiratory pattern in patients who fail weaning from mechanical ventilation. Am. J. Respir. Crit. Care Med..

[91] Albani F., Fusina F., Ciabatti G., Pisani L., Lippolis V., Franceschetti M.E., Giovannini A., di Mussi R., Murgolo F., Rosano A. (2021). Flow index accurately identifies breaths with low or high inspiratory effort during pressure support ventilation. Crit. Care.

[92] Albani F., Pisani L., Ciabatti G., Fusina F., Buizza B., Granato A., Lippolis V., Aniballi E., Murgolo F., Rosano A. (2021). Flow index: A novel, non-invasive, continuous, quantitative method to evaluate patient inspiratory effort during pressure support ventilation. Crit. Care.

[93] Vaporidi K., Akoumianaki E., Telias I., Goligher E.C., Brochard L., Georgopoulos D. (2020). Respiratory drive in critically ill patients. Pathophysiology and clinical implications. Am. J. Respir. Crit. Care Med..

[94] Piquilloud L., Beloncle F., Richard J.M., Mancebo J., Mercat A., Brochard L. (2019). Information conveyed by electrical diaphragmatic activity during unstressed, stressed and assisted spontaneous breathing: A physiological study. Ann. Intensive Care.

[95] Bellani G., Mauri T., Coppadoro A., Grasselli G., Patroniti N., Spadaro S., Sala V., Foti G., Pesenti A. (2013). Estimation of patient’s inspiratory effort from the electrical activity of the diaphragm. Crit. Care Med..

[96] Di Mussi R., Spadaro S., Mirabella L., Volta C.A., Serio G., Staffieri F., Dambrosio M., Cinnella G., Bruno F., Grasso S. (2016). Impact of prolonged assisted ventilation on diaphragmatic efficiency: Nava versus PSV. Crit. Care.

[97] Okahara S., Shimizu K., Morimatsu H. (2017). Severe acute respiratory distress syndrome using electrical activity of the diaphragm on weaning from extracorporeal membrane oxygenation. Acta Med. Okayama.

[98] Mauri T., Grasselli G., Suriano G., Eronia N., Spadaro S., Turrini C., Patroniti N., Bellani G., Pesenti A. (2016). Control of respiratory drive and effort in extracorporeal membrane oxygenation patients recovering from severe acute respiratory distress syndrome. Anesthesiology.

[99] Di Mussi R., Spadaro S., Volta C.A., Bartolomeo N., Trerotoli P., Staffieri F., Pisani L., Iannuzziello R., Dalfino L., Murgolo F. (2020). Continuous assessment of neuro-ventilatory drive during 12 h of pressure support ventilation in critically ill patients. Crit. Care.

[100] Garofalo E., Bruni A., Pelaia C., Landoni G., Zangrillo A., Antonelli M., Conti G., Biasucci D.G., Mercurio G., Cortegiani A. (2019). Comparisons of two diaphragm ultrasound-teaching programs: A multicenter randomized controlled educational study. Ultrasound J..

[101] Goligher E.C., Laghi F., Detsky M.E., Farias P., Murray A., Brace D., Brochard L.J., Bolz S.S., Rubenfeld G.D., Kavanagh B.P. (2015). Measuring diaphragm thickness with ultrasound in mechanically ventilated patients: Feasibility, reproducibility and validity. Intensive Care Med..

[102] Moury P.H., Zunarelli R., Bailly S., Durand Z., Behouche A., Garein M., Durand M., Verges S., Albaladejo P. (2021). Diaphragm thickening during venoarterial extracorporeal membrane oxygenation weaning: An observational prospective study. J. Cardiothorac. Vasc. Anesth..

[103] Cammarota G., Rossi E., Vitali L., Simonte R., Sannipoli T., Anniciello F., Vetrugno L., Bignami E., Becattini C., Tesoro S. (2021). Effect of awake prone position on diaphragmatic thickening fraction in patients assisted by noninvasive ventilation for hypoxemic acute respiratory failure related to novel coronavirus disease. Crit. Care.

[104] Gautier M., Joussellin V., Ropers J., El Houari L., Demoule A., Similowski T., Combes A., Schmidt M., Dres M. (2023). Diaphragm function in patients with COVID-19-related acute respiratory distress syndrome on venovenous extracorporeal membrane oxygenation. Ann. Intensive Care.

[105] Haaksma M.E., Smit J.M., Boussuges A., Demoule A., Dres M., Ferrari G., Formenti P., Goligher E.C., Heunks L., Lim E.H.T. (2022). Expert consensus on diaphragm ultrasonography in the critically ill (exodus): A Delphi consensus statement on the measurement of diaphragm ultrasound-derived parameters in a critical care setting. Crit. Care.

[106] Cammarota G., Sguazzotti I., Zanoni M., Messina A., Colombo D., Vignazia G.L., Vetrugno L., Garofalo E., Bruni A., Navalesi P. (2019). Diaphragmatic ultrasound assessment in subjects with acute hypercapnic respiratory failure admitted to the emergency department. Respir. Care.

[107] Spadaro S., Grasso S., Mauri T., Dalla Corte F., Alvisi V., Ragazzi R., Cricca V., Biondi G., Di Mussi R., Marangoni E. (2016). Can diaphragmatic ultrasonography performed during the t-tube trial predict weaning failure? The role of diaphragmatic rapid shallow breathing index. Crit. Care.

[108] Cammarota G., Boniolo E., Santangelo E., De Vita N., Verdina F., Crudo S., Sguazzotti I., Perucca R., Messina A., Zanoni M. (2021). Diaphragmatic kinetics assessment by tissue doppler imaging and extubation outcome. Respir. Care.

[109] Soilemezi E., Savvidou S., Sotiriou P., Smyrniotis D., Tsagourias M., Matamis D. (2020). Tissue doppler imaging of the diaphragm in healthy subjects and critically ill patients. Am. J. Respir. Crit. Care Med..

[110] Oppersma E., Hatam N., Doorduin J., van der Hoeven J.G., Marx G., Goetzenich A., Fritsch S., Heunks L.M.A., Bruells C.S. (2017). Functional assessment of the diaphragm by speckle tracking ultrasound during inspiratory loading. J. Appl. Physiol. (1985).

[111] Shi Z.H., de Vries H., de Grooth H.J., Jonkman A.H., Zhang Y., Haaksma M., van de Ven P.M., de Man A., Girbes A., Tuinman P.R. (2021). Changes in respiratory muscle thickness during mechanical ventilation: Focus on expiratory muscles. Anesthesiology.

[112] Bruni A., Garofalo E., Pelaia C., Messina A., Cammarota G., Murabito P., Corrado S., Vetrugno L., Longhini F., Navalesi P. (2019). Patient-ventilator asynchrony in adult critically ill patients. Minerva Anestesiol..

[113] Garofalo E., Bruni A., Pelaia C., Liparota L., Lombardo N., Longhini F., Navalesi P. (2018). Recognizing, quantifying and managing patient-ventilator asynchrony in invasive and noninvasive ventilation. Expert. Rev. Respir. Med..

[114] Soilemezi E., Vasileiou M., Spyridonidou C., Tsagourias M., Matamis D. (2019). Understanding patient-ventilator asynchrony using diaphragmatic ultrasonography. Am. J. Respir. Crit. Care Med..

[115] Vivier E., Haudebourg A.F., Le Corvoisier P., Mekontso Dessap A., Carteaux G. (2020). Diagnostic accuracy of diaphragm ultrasound in detecting and characterizing patient-ventilator asynchronies during noninvasive ventilation. Anesthesiology.

[116] Mulqueeny Q., Ceriana P., Carlucci A., Fanfulla F., Delmastro M., Nava S. (2007). Automatic detection of ineffective triggering and double triggering during mechanical ventilation. Intensive Care Med..

[117] Chen C.W., Lin W.C., Hsu C.H., Cheng K.S., Lo C.S. (2008). Detecting ineffective triggering in the expiratory phase in mechanically ventilated patients based on airway flow and pressure deflection: Feasibility of using a computer algorithm. Crit. Care Med..

[118] Gutierrez G., Ballarino G.J., Turkan H., Abril J., De La Cruz L., Edsall C., George B., Gutierrez S., Jha V., Ahari J. (2011). Automatic detection of patient-ventilator asynchrony by spectral analysis of airway flow. Crit. Care.

[119] Younes M., Brochard L., Grasso S., Kun J., Mancebo J., Ranieri M., Richard J.C., Younes H. (2007). A method for monitoring and improving patient: Ventilator interaction. Intensive Care Med..

[120] Sinderby C., Liu S., Colombo D., Camarotta G., Slutsky A.S., Navalesi P., Beck J. (2013). An automated and standardized neural index to quantify patient-ventilator interaction. Crit. Care.

[121] Mauri T., Bellani G., Grasselli G., Confalonieri A., Rona R., Patroniti N., Pesenti A. (2013). Patient-ventilator interaction in ards patients with extremely low compliance undergoing ecmo: A novel approach based on diaphragm electrical activity. Intensive Care Med..

[122] Ramji H.F., Hafiz M., Altaq H.H., Hussain S.T., Chaudry F. (2023). Acute respiratory distress syndrome; a review of recent updates and a glance into the future. Diagnostics.

[123] Battaglini D., Fazzini B., Silva P.L., Cruz F.F., Ball L., Robba C., Rocco P.R.M., Pelosi P. (2023). Challenges in ARDS definition, management, and identification of effective personalized therapies. J. Clin. Med..

